# Termination of atrial arrhythmia and restoration of sinus rhythm during pulsed field ablation with a pentaspline catheter in patients with persistent atrial fibrillation

**DOI:** 10.1093/europace/euaf144

**Published:** 2025-07-08

**Authors:** Massimo Moltrasio, Saverio Iacopino, Francesco Solimene, Stefano Bianchi, Marco Schiavone, Sakis Themistoclakis, Antonio Rossillo, Matteo Bertini, Davide Zirolia, Mario Volpicelli, Gianluca Zingarini, Antonio Dello Russo, Maurizio Malacrida, Giulio Zucchelli, Claudio Tondo

**Affiliations:** Department of Clinical Electrophysiology & Cardiac Pacing, Monzino Cardiological Center IRCCS, Via Carlo Parea 4, Milan 20138, Italy; Maria Cecilia Hospital, GVM Care & Research, Cotignola, Italy; Electrophysiology Unit, Montevergine Clinic, Mercogliano, AV, Italy; Department of Biomedical Sciences and Public Health, Marche Polytechnic University, Ancona, Italy; Center of Excellence in Cardiovascular Sciences, Ospedale Isola Tiberina—Gemelli Isola, Rome, Italy; Department of Clinical Electrophysiology & Cardiac Pacing, Monzino Cardiological Center IRCCS, Via Carlo Parea 4, Milan 20138, Italy; Cardiology Department, Ospedale dell’angelo, Mestre, Venice, Italy; Cardiology Unit, San Bortolo Hospital, Vicenza, Italy; Cardiology Unit, University of Ferrara, Sant’Anna University Hospital, Ferrara, Italy; Clinical and Experimental Cardiology, Clinical and Interventional Cardiology, University Hospital, Sassari, Italy; Cardiovascular Diseases and Electrophysiology Unit, S. Maria della Pietà Hospital, Nola, Naples, Italy; Cardiology Unit, S. Maria della Misericordia Hospital, Perugia, Italy; Department of Biomedical Sciences and Public Health, Marche Polytechnic University, Ancona, Italy; Cardiology and Arrhythmology Clinic, Marche University Hospital, Ancona, Italy; Boston Scientific, Milan, Italy; Second Division of Cardiology, Cardiac-Thoracic-Vascular Department, New Santa Chiara Hospital, Azienda Ospedaliero Universitaria Pisana, Pisa, Italy; Department of Clinical Electrophysiology & Cardiac Pacing, Monzino Cardiological Center IRCCS, Via Carlo Parea 4, Milan 20138, Italy; Department of Biomedical, Surgical and Dental Sciences, University of Milan, Milan, Italy

**Keywords:** Atrial fibrillation, Pulsed field ablation, Cellular electroporation, Sinus rhythm restoration, Persistent AF

## Abstract

**Clinical trial registration**: Advanced TecHnologies For SuccEssful AblatioN of AF in Clinical Practice (ATHENA). URL: http://clinicaltrials.gov/Identifier: NCT05617456.

Restoration of sinus rhythm (SR) solely through ablation is a rarely utilized endpoint in radiofrequency ablation (RFA) for persistent atrial fibrillation (persAF), due to its inconsistent association with long-term outcomes.^1,2^ Moreover, termination of ongoing AF and restoration of sinus rhythm (TASR) often requires extensive substrate modification to affect AF organization.^3^ The clinical relevance of TASR without electrical cardioversion (ECV) as a procedural endpoint remains poorly defined in pulsed field ablation (PFA). The improved safety profile of PFA, due to its nonthermal mechanism enabling broader ablation with minimal collateral damage—particularly to structures like the oesophagus and phrenic nerve^4,5^—may support more extensive ablation strategies and potentially reduce the critical mass of left atrial myocardium required to sustain AF.

Therefore, our objective was to evaluate the clinical significance of TASR in patients with persAF treated with the Farapulse™ system (Boston Scientific), and to identify factors associated with TASR. Consecutive patients with persAF undergoing ablation with the pentaspline PFA catheter at 18 centres were enrolled in the ATHENA registry. Protocol-directed pulmonary vein isolation (PVI) was performed using eight applications per vein, with additional applications delivered to non-PV structures according to operators’ discretion.

Data are available from the corresponding author upon reasonable request. Baseline data and procedural outcomes have been summarized in *Figure 1*. A total of 335 patients with persAF were included in this analysis, 28.1% (*n* = 94) of whom had long-standing persAF (LSPAF). Non-PV ablation was performed in 60.6% (*n* = 203) of cases. Among these, 20% (*n* = 67) were already in SR at the end of the procedure due to prior ECV, 3.3% (*n* = 11) began in AF and remained in AF at the end of the procedure, while 76.7% (*n* = 257) began in AF and subsequently achieved TASR during the procedure. Of those, 33.1% (*n* = 85/257) restored SR directly through PFA energy delivery without the need for ECV. Multivariate analysis indicated that LSPAF was significantly associated with a lower likelihood of TASR (HR = 0.48, *P* = 0.0195). During a median follow-up of 361 [274–415] days, the rate of AF recurrence did not significantly differ between patients who achieved TASR and those who did not (14.2% vs. 24.4%, *P* = 0.0729). However, a non-significant trend towards reduced AF recurrence was observed in TASR patients (HR = 0.55, *P* = 0.0657). Interestingly, recurrence of any atrial arrhythmia was significantly lower in TASR patients (82.4% vs. 70.9%, *P* = 0.0491), suggesting that procedural SR restoration may have some prognostic relevance.

**Figure 1:**
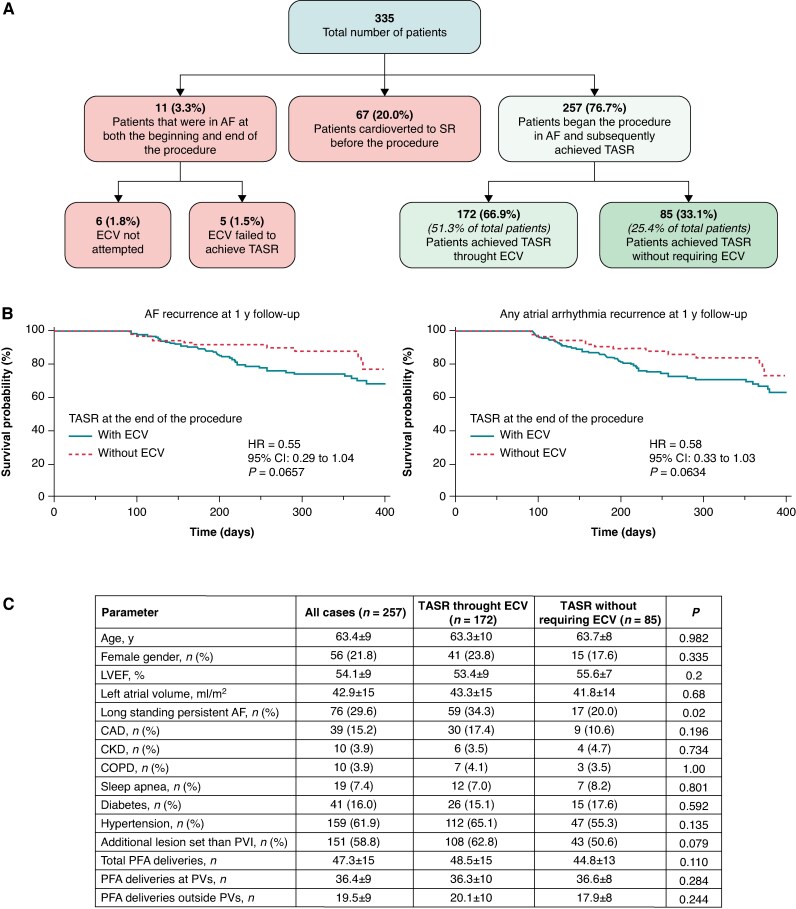
(*A*) Flow diagram summarizing the rhythm outcomes of 335 patients with persistent atrial fibrillation (persAF) undergoing pulsed field ablation (PFA). Patients were grouped based on their rhythm at the beginning and end of the procedure. A total of 257 patients (76.7%) began the procedure in AF and subsequently achieved termination of atrial arrhythmia and sinus rhythm restoration (TASR). Of these, 172 (66.9%) required electrical cardioversion (ECV), while 85 (33.1%) achieved TASR directly through PFA energy delivery without ECV. (*B*) Kaplan–Meier curve showing freedom from AF recurrence at 1-year follow-up in patients who achieved TASR with or without ECV. There was a non-significant trend towards lower recurrence in the group that achieved TASR without ECV (HR = 0.55, 95% CI: 0.29–1.04, *P* = 0.0657). (*C*) Kaplan–Meier curve showing freedom from any atrial arrhythmia recurrence at 1-year follow-up in patients who achieved TASR with or without ECV. A similar trend was observed, favouring patients who restored sinus rhythm without ECV (HR = 0.58, 95% CI: 0.33–1.03, *P* = 0.0634).

This hypothesis is supported by mechanistic data from Haïssaguerre *et al.*^6^ who showed that termination of persistent AF during stepwise catheter ablation was achievable when critical atrial structures were targeted—structures later demonstrated to be suitable for empirical ablation.^7^ In that study, AF termination was preceded by significant cycle length prolongation, most notably after ablation of the PV–LA junction, left atrial appendage, and coronary sinus—common sites of focal atrial tachyarrhythmias (ATs) and frequent non-PV triggers in persistent AF.^8^ Pulsed field ablation’s ability to safely and comprehensively ablate these structures may allow for more effective substrate debulking and increase the likelihood of SR restoration. Acute TASR seen in many patients in this series supports further evaluation of its clinical relevance.

These findings can be better understood in the context of prior RFA studies. Elayi *et al.*^2^ conducted a prospective study of 306 patients undergoing RFA for LSPAF. While procedural termination was shown to predict the mode of recurrence (e.g. patients converting to AT were more likely to experience AT recurrence), it was not associated with improved long-term SR maintenance. An important insight from Elayi’s study was that termination of focal ATs during ablation was associated with significantly better long-term success compared to macroreentrant ATs (83% vs. 57% success, *P* = 0.026). This suggests that TASR may only carry prognostic value when it reflects interruption of focal mechanisms. In contrast, O’Neill *et al.*^1^ reported that procedural AF termination was predictive of superior long-term SR maintenance. Similar findings were reported by Deisenhofer *et al.*^9^ who evaluated patients with drug-refractory persAF randomly assigned to a tailored ablation procedure targeting areas of spatio-temporal dispersion in addition to PVI or PVI-only strategy. Although AF termination during the index procedure in the tailored arm had no impact on the rate of AF recurrence at 12 months, it significantly improved the rate of freedom from any atrial arrhythmia. This difference may result from a stepwise approach using extensive lesions, enabling more complete atrial substrate ablation and arrhythmia termination by confirming isolation through connections to inert structures or adjacent ablated areas. By comparison, the strategy used in Elayi’s study—PVI followed by CFAE ablation—was less reliant on linear lesion sets, which may explain the diminished predictive value of termination. These divergent findings underscore the complex and context-dependent role of procedural termination as a marker of outcome. While O’Neill’s and Deisenhofer’s data suggest that termination can be an endpoint under specific ablation paradigms, Elayi’s work points to the need for mechanistic specificity—particularly the value of focal AT termination—in interpreting TASR. To further complicate matters, PFA operates via a minimal-thermal, electroporation-based mechanism, which may influence substrate modification differently from RFA. This distinction could affect both the pattern of arrhythmia termination and the durability of procedural success, although electrogram characteristics in ablation termination may also play an important role.^10^

Limitations of our study include its observational design, lack of a control group, and focus on a single PFA system. Moreover, given the observational nature of this registry and the absence of a standardized lesion set beyond PVI, operator discretion in additional ablation likely introduced variability in procedural outcomes.

While ATHENA represents one of the largest multicentre PFA experiences to date, randomized studies are needed to determine whether TASR—especially without ECV—should be a procedural endpoint in PFA workflows.

In conclusion, TASR without ECV was achieved in ∼30% of persAF patients undergoing PFA with the Farapulse™ system. Although not independently predictive of long-term freedom from AF, TASR was associated with a reduced risk of any ATs recurrence during follow-up. Therefore, TASR could be explored as a procedural endpoint in future workflows, especially in the context of electroporation-based substrate modification, although this remains to be confirmed in randomized trials.
